# Validity and reliability of the multidimensional health locus of control scale for college students

**DOI:** 10.1186/1471-2458-7-295

**Published:** 2007-10-18

**Authors:** Mahdi Moshki, Fazlollah Ghofranipour, Ebrahim Hajizadeh, Parviz Azadfallah

**Affiliations:** 1 Dept. Health, Gonabad University of Medical Sciences, Gonabad, Khorasan Razavi, I.R.Iran; 2 Dept. Health Education, School of Medical Sciences, Tarbiat Modares University, Tehran, I.R.Iran; 3 Dept. Biostatistics, School of Medical Sciences, Tarbiat Modares University, Tehran, I.R.Iran; 4 Dept. Psychology, School of Humanities Sciences, Tarbiat Modares University, Tehran, I.R.Iran

## Abstract

**Background:**

The purpose of the present study was to assess the validity and reliability of Form A of Multidimensional Health Locus of Control scales in Iran. Health locus of control is one of the most widely measured parameters of health belief for the planning of health education programs.

**Methods:**

496 university students participated in this study. The reliability coefficients were calculated in three different methods: test-retest, parallel forms and Cronbach alpha. In order to survey validity of the scale we used three methods including content validity, concurrent validity and construct validity.

**Results:**

We established the content validity of the Persian translation by translating (and then back-translating) each item from the English version into the Persian version. The concurrent validity of the questionnaire, as measured by Levenson's IPC scale was .57 (P < .001), .49 (P < .01) and .53 (P < .001) for IPC, respectively. Exploratory principal components analysis supported a three-factor structure that items loading adequately on each factor. Moreover, the approximate orthogonal of the dimensions were obtained through correlation analyses. In addition, the reliability results were acceptable, too.

**Conclusion:**

The results showed that the reliability and validity of Persian Form A of MHLC was acceptable and respectable and is suggested as an applicable criterion for similar studies in Iran.

## Background

Health Locus of Control (HLC, hereafter) is one of the most commonly-used parameters of health belief in planning the health education programs. In fact, the HLC is the degree to which individual believe that his or her behavior is controlled by external or internal factors [1,2].

The Multidimensional HLC scales have been used as one of the most efficient measures of health-related beliefs for more than a quarter of a century. HLC has been recognized as an important construct in understanding and predicting health behaviors [3]. It has helped to shape our thinking about the role of beliefs in the context of health behaviors, health outcomes and health care. According to Rotter's (1966) social learning theory, individuals may have internal or external locus of control, often abbreviated as I/E dimension [4,5].

Wallston with their colleagues (1978) deserves the acclaim to have applied successfully Rotter's basic idea to the health domain. The term 'locus' refers to the location where control resides either 'internal' to the individual who believe certain events and happenings are due to their own actions and behaviors, that is, their own actions are directly responsible for their events in their lives or 'external' to the individual who believe certain events and happenings in their lives are due to factors such as physicians, chance, fate, and luck. They developed a unidimensional HLC scale and began using it in studies in the 1970s [5,6]. The results from the early studies with the unidimensional HLC Scale convinced the Wallstons that internality and externality are separate dimensions. Following Levenson's (1974) splitting Rotter's I-E construct into three dimensions – Internal, Powerful Others, and Chance – they developed the Multidimensional Health Locus of Control (MHLC) scales. The MHLC scales consist of two equal and parallel forms, A & B that are the 'general' health locus of control scales [6]. Wallston, Stein, & Smith (1994) developed Form C of the MHLC in which they split the Powerful Others dimension into two subscales: Doctors and Other People [7]. Finally, Wallston et al. (1999) added a new subscale assessing beliefs about God as a locus of control of one's health status [8]. Chaplin et al., (2001) used factor analysis for this four-factor scale. Their findings showed that despite a desirable correlation between the three external factors of God, Powerful Others, and Chance, the four-factor condition which takes into account an internal control factor yields the best outcome. And these sub-scales of MHLC can be scored separately as different dimensions [9].

The HLC is regarded as an effective variable in the development of health behavior, clinical capacity, and determining the health problems. The Internal HLC is positively accompanied with knowledge and attitude, psychological state, health behavior, and better health conditions. On the other hand, most of the external HLC is accompanied with negative health behaviors and weak psychological state [3]. As such, various scales of HLC have been developed in general populations or children. And many studies have been conducted on this scale throughout the world which has led to valuable outcomes in the field of health psychology. The findings of these studies can be found in over 380 articles available in different data bases such as Academic Search Premier, Medline, Eric, and Health Source: e.g., Nursing/Academic Edition, American Humanities Index, Health Source Consumer Edition and Psych Articles [10]. In the past 25+ years, Form A has been used in over a thousand studies and has been cited in literature hundreds of times [5].

Comparison Iranian health belief and especially general health data with Western data using a common scale such as Form A of the MHLC is also indispensable in order to grasp any features that are characteristics of Iranian samples. Such an approach would provide insight into the general health of the Iranian community setting, so that we could choose or modify a number of health education and promotion projects. Despite its importance, the validity and reliability of the MHLC scale for Iranian population have not yet been verified. In the first step, the present study assessed the psychometric characteristics of Form A among Iranian college student. In so doing, various kinds of validity, namely, content validity, concurrent validity, and construct validity of this scale will be determined. Then, the reliability of the scale will be examined through test-retest, parallel forms, and internal consistency methods of estimating reliability. And finally, some suggestions and implications for further research will be put forward.

## Methods

### Ethical considerations

Ethical approval of this study was gained from the Research Ethics Committee, which at the time was based at Tarbiat Modares University.

### Measures

Form A of the MHLC scales include 18 items and consist of three subscales, namely Internal Health Locus of Control, Powerful Others Health Locus of Control, and Chance Health Locus of Control. Each of these subscales contains six items with a six-point Likert response scale ranging from 'Strongly Agree' to 'Strongly Disagree'. Scales are scored by summing respective items for a total scale score. Higher scores reflect stronger endorsement of MHLC scales [6]. Internal HLC refers to the extent that personal behavioral factors are responsible for one's health or illness; Powerful Others HLC encompasses the degree to which one's health is influenced by others for example, by physicians or other healthcare professionals; and Chance HLC taps one's belief that his health depends on chance, luck, or fate.

In order to assess concurrent validity we used the Form A of MHLC and Levenson's IPC scales (Persian language versions) simultaneously. The Levenson's IPC scale, which is a six- point Likert scale type, includes twenty-four items that similar to MHLCS it includes these components: internal, powerful others, and chance. Each one consists of eight questions, which measure individuals' belief level in the cases that were reminded before. Validity of IPC scale has been determined with Rotter's I-E scale (1996). Also Levenson has reported Kuder-Richardson's reliability coefficient for every scales of IPC 0.50, 0.61 and 0.77 respectively. The validity and reliability of Farsi manuscript of this scale was reported by Farahani, Cooper, & Jin (1996). For example, reliability coefficients for I, P and C were respectively 0.76, 0.56 and 0.67 among students [11].

### Procedure

The 'forward-backward' procedure was applied to translate Form A of the MHLC from English into Persian. The original 18 items questionnaire was translated into Persian by the authors, and then was translated back into English by two bilinguals who were blind to the original English version. The expert panel (majoring in psychology, specialist in Persian language and health sciences) reviewed our back- translation and some corrections were made accordingly. After that, in a pilot study, the edited version of the questionnaire was submitted to a group of 130 students from Tehran Medical University. There were two purposes for this review: first, to ascertain whether the student's understanding of the questionnaire items was the same as that of the researcher; and second, whether there was any disagreement among the students regarding their understanding of the items. Afterwards, the students' comments were taken into account and some modifications were done where necessary. Questionnaires were filled out by a group of college students. Students completed the paper-and-pencil measures in a classroom setting, which was staffed by research assistants who were available to answer the questions if necessary.

### Participants

The participants of this study were 496 college students studying in different courses of medical sciences from Tehran and Gonabad Medical Universities. All of the samples participated willingly and voluntarily in this study and so all respondents in the sample completed the questionnaires in full.

### Analyses

To examine the psychometric characteristics of the Persian version of Form A of the MHLC scales, the following analyses were performed through the Statistical Package for Social Sciences (SPSS) version 11.5 and STATISTICA soft ware. To establish the validity of the scale, the content validity was determined through Persian translation by faithfully translating (and then back- translating) each item from the English version into the Persian version; the concurrent validity was determined through the concurrent administration of this scale with the Persian language version of the Levenson's IPC scale; the construct validity was examined through exploratory factor analysis (EFA) and the minimum loading employed to retain in each factor was 0.40.

In regard to reliability, test-retest method and parallel forms were applied using Pearson Product moment correlation. The internal consistency of the scale was measured through Cronbach's coefficient α. According to Wallston [12] modest reliability which is ranging from 0.60 to 0.75, is acceptable in the research.

## Results

It should be mentioned that the 496 students, 30.2 percent of whom were male and 69.8 percent were female were randomly selected from all the above courses. They had a mean age of 20.6 years (SD = 2.05). Also, descriptive information for MHLC and Levenson's I, P, & C scales is included in Tables 1 and 2.

**Table 1:** Descriptive data on scales

***Scale***	**Item**	**Mean**	**SD**
*Form A*			
IHLC	6	23.53	4.59
PHLC	6	20.12	7.60
CHLC	6	14.36	7.81
*Levenson*			
I Scale	8	35.30	4.32
P Scale	8	21.78	7.18
C Scale	8	19.60	7.12

The means and standard deviations of the scales gained after doing the concurrent validity.

**Table 2:** Mean values, variances, correlations, and cronbach's alpha coefficients of items of Form A

	***Mean***	***Variance***	***Corrected item-total correlation***	***Alpha if item Deleted***	***Standardized alpha of the subscale***
Item 1	23.442	10.529	0.302	0.680	0.68
Item 6	23.680	8.916	0.487	0.625	(Internal)
Item 8	24.245	9.212	0.356	0.678	
Item 12	23.278	10.070	0.548	0.619	
Item 13	23.508	9.855	0.460	0.635	
Item 17	23.073	10.382	0.465	0.640	
Item 3	19.904	14.557	0.494	0.670	0.72
Item 5	20.829	14.462	0.406	0.703	(Powerful others)
Item 7	19.723	14.661	0.522	0.662	
Item 10	20.446	14.922	0.468	0.678	
Item 14	20.287	16.922	0.384	0.703	
Item 18	19.553	15.692	0.475	0.678	
Item 2	14.677	16.457	0.263	0.601	0.66
Item 4	13.828	16.658	0.316	0.576	(Chance)
Item 9	14.126	16.522	0.509	0.638	
Item 11	14.387	13.925	0.658	0.438	
Item 15	14.387	13.925	0.658	0.438	
Item 16	14.806	16.256	0.364	0.588	

The means calculated by eliminating the item from Form A.

### Content validity

To examine the content validity of the translated version of MHLC, as a mentioned in Procedure section, we established by faithfully translating (and then back- translating) each item from the English version into the Persian version.

### Concurrent validity

In the concurrent administration of the scales, the participants were divided into two equal groups randomly in such a way that half of the participants first answered the translated Form A then Persian version of the Levenson's IPC scale; and the other half first answered Levenson's questionnaire then Form A. This was done to control the test effect. The obtained results indicated significant correlation coefficients between the two scale factors i.e., 0.57 for Internal (P < 0.001), 0.49 for Powerful Others (P < 0.01), and 0.53 for Chance (p < 0.001).

### Construct validity

#### Exploratory factor analysis (EFA)

Three factors were found by principal component exploratory factor analysis with criterion of Kaiser's eigenvalue above one. The eigenvalues were 4.93, 3.32, and 2.34 for factors one, two and three, respectively. The three significant factors could account for 58.90 percent of the total variance and the variances explained of the test were 27.43, 18.46, and 13.00 percent for the three factors. The factors extracted in the student sample corresponded very closely to the theoretical constructs. Items 1, 6, 8, 12, 13, and 17 from the original Internal scale loaded on factor one. Items 3, 5, 7, 10, 13, and 18 from the original Powerful Others scale loaded on factor two. Items 2, 4, 9, 11, 15, and 16 from the original Chance scale loaded on factor three (Table 3).

**Table 3:** Factor analysis of the Form A items using varimax rotation method

	***IHLOC***	***PHLOC***	***CHLOC***
	
***Item***	***F1***	***F2***	***F3***
1	0.63		
2	-0.41		0.55
3		0.54	
4	-0.59		0.42
5		0.71	-0.42
6	0.61		
7		0.62	
8	0.82		
9			0.74
10		0.78	
11			0.78
12	0.54		
13	0.72		
14		0.67	
15			0.69
16			0.40
17	0.69		
18		0.43	

Items with factor loading < 0.40 are not listed.

#### Correlation analysis

For bivariate correlation among the subscales correlation analysis was calculated. In this regard, there was a positive but weak correlation (0.28) between the Internal HLC and Powerful HLC, no correlation was found between the Chance HLC and Powerful Others HLC (r = -0.31); and a negatively weak correlation coefficient was found between the Internal HLC and the Chance HLC (r = -0.20).

## Reliability

### Test -retest

To determine the reliability of this scale, 496 students answered the questionnaire items, and after a time interval of 4 weeks, the questionnaire was administered again. The reliability indices for the Internal, Chance, and Powerful Others using Pearson's moment correlation were 0.60 (p < 0.001), 0.58 (p < 0.002), and 0.74 (p < 0.0001), respectively (Table 4). These levels reported sufficient test- retest stability coefficients by Wallston [12].

**Table 4:** Paired samples correlations of Form A by using test-retest

***Pair***	***N***	***Correlation***	***Significance***
Internal 1&2	496	0.601	0.001
Chance 1&2	496	0.587	0.002
Powerful 1&2	496	0.746	0.000

The paired samples obtained after two administrations of the scale.

### Parallel forms

The original form of MHLC was administered to 30 senior English students. Then, the same group took the translated version of MHLC after one week. Using the spilt-half method and Spearman Brown Prophecy formula, the correlation coefficient between the Persian version and the original form of MHLC were estimated as 0.71 for Internal HLC, 0.70 for Chance HLC, and 0.72 for Powerful Others HLC. All of which were significant (p < 0.0001). Comparisons between Persian and English version of Form A showed a sufficient consistent.

### Internal consistency

Cronbach's coefficient α was employed to estimate the internal consistency of the scale that is reported in Table 2. Cronbach's alpha coefficients were moderately acceptable for Internal (0.68), for Powerful Others (0.72), and for Chance (0.66).

## Discussion

Form A of MHLC scales has been employed in many studies throughout the world due to its easy administration, objectivity, and having appropriate psychometric characteristics, especially in the health domain, promoting of health and health psychology. Using Form A will allow assessing subjects' general health locus of control beliefs but researcher won't always be informed that what this means to his/her subjects.

The obtained results indicated that the reliability of Form A was rather acceptable. Cronbach's coefficient alphas for Internal, Powerful Others, and Chance factors were comparable to that of Wallston's normative data [6,12] and so were in accordance with the findings (ranging 0.54–0.78), of Winefield [13]; Marshal and colleagues [14], Tanabe [15], Blay and Astrom [16], Rodriguez-Rosero and colleagues [17], Birkett [18], Kuwahara and colleagues [2], Malcarne and colleagues [19].

Considering the validity of Form A, like other studies in different countries, the content, concurrent, and construct kinds of validity were taken into consideration. Regarding the concurrent validity, the obtained results between Form A of MHLC of Levenson and the Persian version are analogous to the reports of Wallston and colleagues [6] and Wallston [12]. He stated that there was a significant correlation between the sub-scales of MHLC and their counterparts in Levenson's Internal, Powerful Others, and Chance subscale. For making sure of the quality of the study, concurrent validity was estimated according to Gold Standard model [6,12].

The three extracted factors in the student sample corresponded very closely to the theoretical constructs of Form A. The results were similar to the findings of Hartke and Kunce, 1982; Russel and Ludenia, 1983; Wall et al., 1989; Marshal et al., 1990; Robinson-Whelen and Storandt, 1992; Casey et al., 1993; and Talbot et al., 1996 [20]. On the other hand, as Wallston [10,20] stated some studies (e.g. Boyle and Harrison, 1981; Meyers et al., 1982; O'Looney and Barrett, 1983; Gutkin et al., 1985; Cooper and Fraboni, 1990; Rogers, 1995; Menzoine et al, 2003) have proposed two simpler factors (Internal versus External) in this scale. Further, Luszczynska and Schwarzer [10] mentioned that Maclachlan and colleagues in 1986 did not come up factorial structure of MHLC among Malawi students but distinguished three factors related to the limitations of medical care and its effect on health. Also, in another study by Astrom and Blay [16], they found a two-factor structure among the adolescents in Ghana.

Furthermore, the correlation coefficients between the sub-scales pointed out that the sub-scales of Internal, Powerful Others, and Chance were orthogonal to one another, a finding which is similar to that of Wallston and colleagues [12]. He mentioned that "the IHLC and PHLC subscales were uncorrelated with one another, PHLC and CHLC were only weakly positively correlated, and IHLC and CHLC were weakly negatively intercorrelated. Thus supporting the construct validity claims that these dimensions were more-or-less orthogonal to one another".

In another study conducted in New Zealand a much more degree of correlation among the factors was found [10].

In conclusion, the findings of this study indicated that the Persian version of Form A is reliable and valid for using in studies of health beliefs in Iranian language countries.

## Limitations and suggestions

In this study, the researchers could not take into account some factors such as socio-economic status for difficulty in obtaining such information from the students, and at the same time it is an important socio-cultural variable which can help us in the explanation and interpretation of MHLC beliefs. In order to assess concurrent validity, we used Persian version of the Levenson's IPC scale which its validity and reliability was done among university students of non-medical courses. Also, there is not a suitable of the Persian version scale for convergent. It should be mentioned that the subjects of the current study were highly educated.

It would be desirable to examine the validity and reliability of Form A in a national sample. Further studies should test the cross-cultural and external validity of Form A in a broader range of samples. Moreover, the Persian version of Form A can be validated to predict health-related behavior or health status. It is also suggested that the validity and reliability of Forms B, C and God be assessed in Iran. Qualitative and quantitative studies of health locus of control constructs are needed to address whether expansion or modification of the MHLC is needed for Iranian ethnic groups. Further, this scale can be administered to different groups of unhealthy people.

## Conclusion

In spite of all the limitations, this study is the first to have examined the validity and reliability of MHLC scale in Iranian subjects.

## Competing interests

The author(s) declare that they have no competing interests.

## Authors' contributions

All authors read and approved the final manuscript and contributed equally to this research

## Pre-publication history

The pre-publication history for this paper can be accessed here:



## References

[B1] Wallston KA, Steptoe A, Apples A (1989). Assessment of control in health-care settings. Stress, Personal Control and Health.

[B2] Kuwahara A, Nishino Y, Ohkubo T, Tsuji I, Hisamichi S, Hosokawa T (2004). Reliability and validity of the multidimensional health locus of control scale in Japan: relationship with demographic factors and health- related behavior. Tohoku J Exp Med.

[B3] Malcarne VL, Drahota A, Hamilton NA (2005). Children's health- related locus of control beliefs: ethnicity, gender, and family income. Child Health Care.

[B4] Health Locus of Control. http://www.hsc.usf.edu.

[B5] Multidimensional health locus of control (MHLC) scales. http://www.vanderbilt.edu.

[B6] Wallston KA, Wallston BS, DeVellis R (1978). Development of the multidimensional health locus of control (MHLC) scales. Health Educ Monogr.

[B7] Wallston KA, Stein MJ, Smith CA (1994). Form C of the MHLC scales a condition – specific measure of locus of control. J Pers Assess.

[B8] Wallston KA (1999). Does God determine your health? The God locus of health control scale. Cognit Ther Res.

[B9] Baken D, Stephens Ch (2005). More dimensions for the multidimensional health locus of control: confirmatory factor analysis of competing models of the structure of control beliefs. J Health Psychol.

[B10] Luszczynska A, Schwarzer R (2005). Multidimensional health locus of control: comments on the construct and its measurement. J Health Psychol.

[B11] Farahani M, Cooper M, Jin P (1996). Is locus of control unidimensional or multidimensional? – Data from Persian translations of Rotter's I-E scale and Levenson's I, P, and C scales. Psychol Res.

[B12] Wallston KA (2005). The validity of multidimensional health locus of control scales. J Health Psychol.

[B13] Winefield HR (1982). Reliability and validity of health locus of control scale. J Pers Assess.

[B14] Marshall GN, Collinns BE, Crooks VC (1990). A comparison of two multidimensional health locus of control instruments. J Pers Assess.

[B15] Tanabe K (1997). Validity and reliability of the children's multidimensional health locus of control scales. Nihon Kango Kagakkaishi.

[B16] Astrom AN, Blay D (2002). Multidimensional health locus of control scales: applicability among Ghanaian adolescents. East Afr Med J.

[B17] Rodriguez-Rosero JE, Ferriani MG, Dela Coleta MF (2002). Multidimensional health locus of control scale – MHLC: validation study. Rev Lat Am Enfermagem.

[B18] McMaster University. selecting the number of response categories for a likert-type scale. http://www.google.com/advanced_search.

[B19] Malcarne VL, Fernandes S, Flores L (2005). Factorial validity of multidimensional health locus of control scales for three American ethnic groups. J Health Psychol.

[B20] Wallston KA (2005). Overview of the special issue on research with the multidimensional health locus of control (MHLC) scales. J Health Psychol.

